# Supplementary material for: DMRforPairs: identifying Differentially Methylated Regions between unique samples using array based methylation profiles
Source: BMC Bioinformatics. 2014 May 15;15:141. doi: 10.1186/1471-2105-15-141 (PMC4046028; doi:10.1186/1471-2105-15-141)
Supplement: Additional file 2 — DMRforPairs output for the comparison of A431-MCF7 and NA17018-NA17105. Please start from the HTML files in each folder. Available via the BMC Bioinformatics website. [file 1471-2105-15-141-S2.zip › 1394847754114233_MOESM2_ESM/A431_MCF7/figures/17149.pdf]

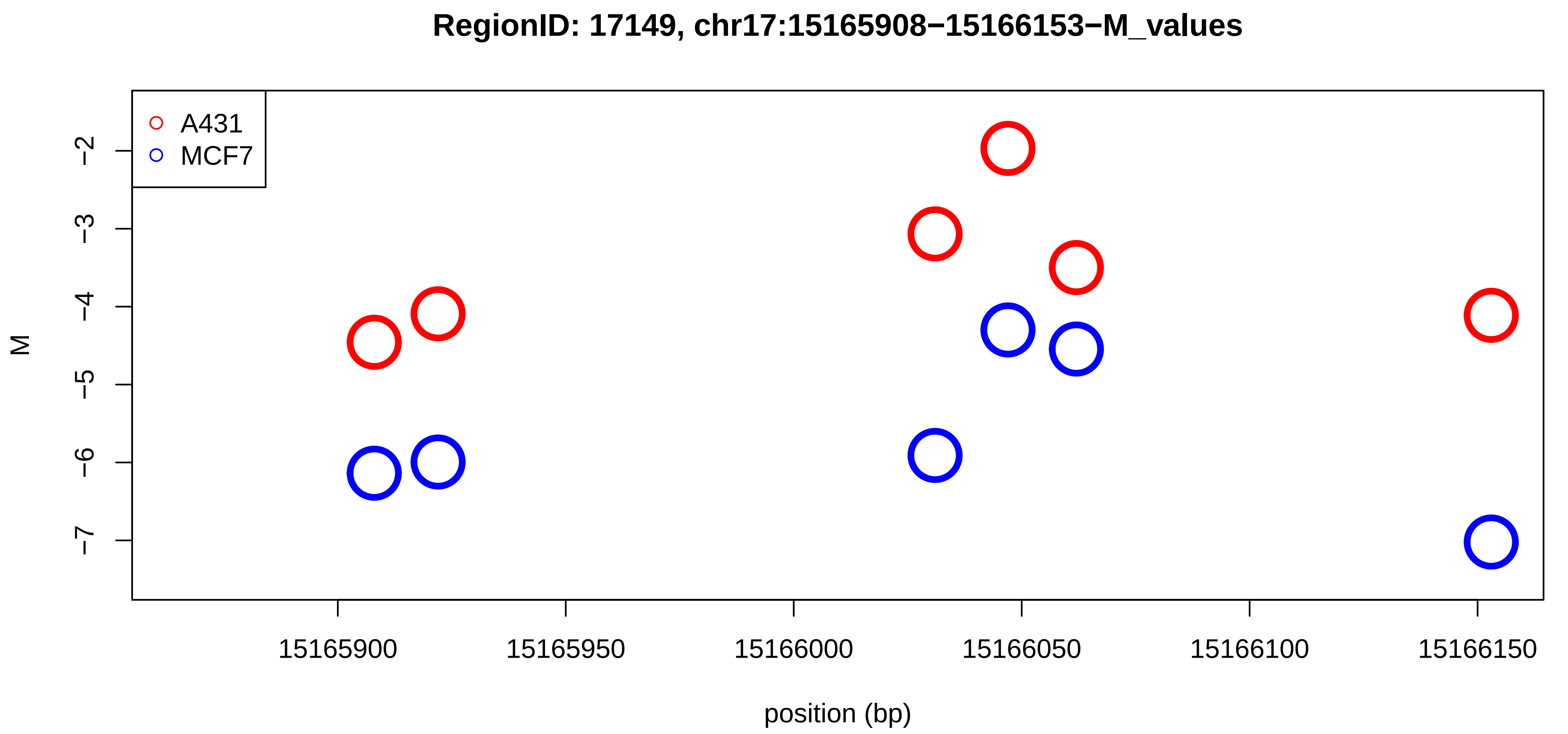

RegionID: 17149, chr17:15165908−15166153−M_values
A431
MCF7
M
−2
−3
−4
−5
−6
−7
15165900
15165950
15166000
15166050
15166100
15166150
position (bp)

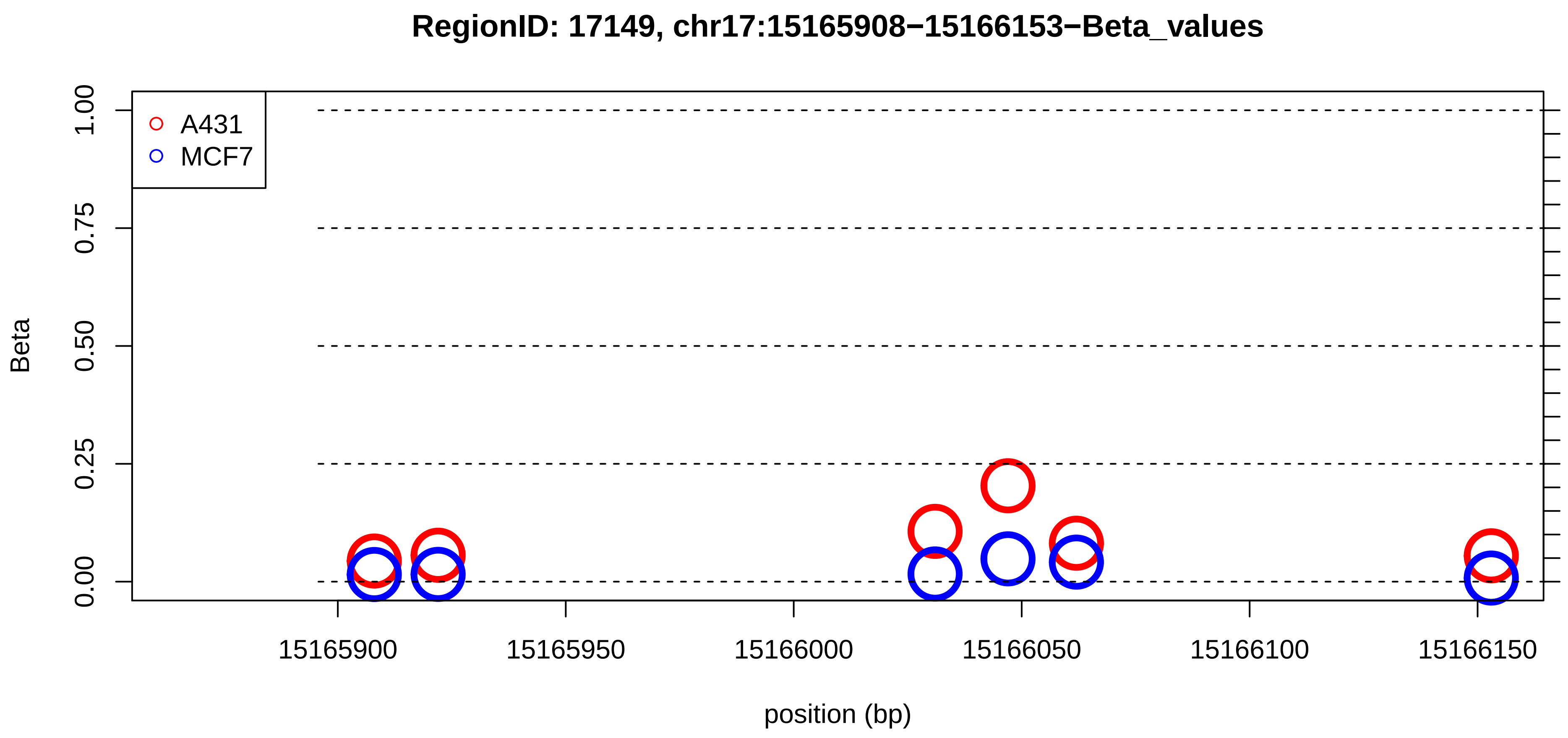

RegionID: 17149, chr17:15165908−15166153−Beta_values
A431
MCF7
Beta
1.00
0.75
0.50
0.25
0.00
15165900
15165950
15166000
15166050
15166100
15166150
position (bp)

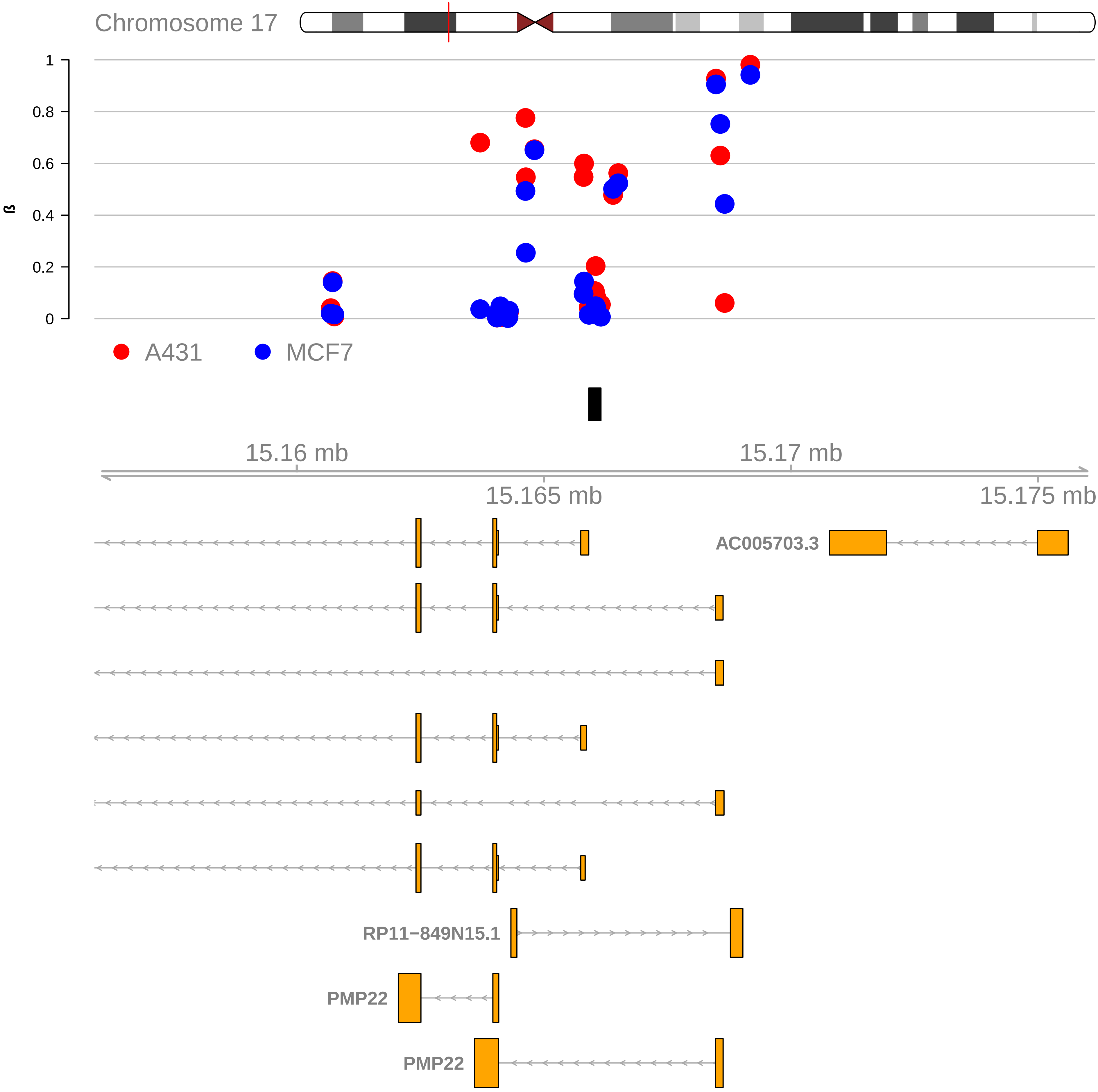

Chromosome 17
1
0.8
0.6
0.4
0.2
0
ß
A431
MCF7
15.16 mb
15.17 mb
15.165 mb
15.175 mb
AC005703.3
RP11−849N15.1
PMP22
PMP22
